# Language-Switching Cost in Chinese-English Bilingual Reading Comprehension: Evidence from Eye-Tracking

**DOI:** 10.1007/s10936-026-10315-z

**Published:** 2026-10-05

**Authors:** Danyi Chen, Kiel Christianson

**Affiliations:** 1 Department of Educational Psychology, University of Illinois, Urbana-Champaign, USA; 2 Beckman Institute for Advanced Science and Technology, Urbana, USA

## Abstract

Code-switching, or language-switching, which is the use of elements from two or more languages in the same utterance or in the same stretch of conversation (Paradis et al., 2011), is prevalent among bilingual and multilingual speakers. According to the parallel activation hypothesis of language comprehension, both languages of a bilingual are activated during sentence processing, even when there is only monolingual input (Spivey & Marian, 1999). However, studies have also shown that such language mixing can slow down processing (Macnamara & Kushnir, 1971; Soares & Grosjean, 1984). This cost has been attributed to either the difficulty of switching languages or the appearance of an unexpected word or wordform. This eye-tracking experiment investigated the cognitive cost brought by code-switching in Chinese-English bilinguals. Specifically, we sought to determine the source of the baseline cost of language-switching in inter-sentential code-switching. Results suggest that narrative consistency and the amount of previous information had longer-lasting effects on bilinguals’ total reading times, along with a brief disruption caused by unexpected wordforms. Taken together, the results indicate that the cognitive cost of language-switching arises more from the surprisal of encountering an unpredicted word or wordform, rather than encountering an unexpected language.

**Supplementary Information:**

The online version contains supplementary material available at https://doi.org/10.1007/s10936-026-10315-z.

## Literature Review

Code-switching, or the use of elements from two or more languages in the same utterance or in the same stretch of conversation (Poplack, 1980; Paradis et al., 2011), is prevalent in bilingual and multilingual speaker production (speech). The switch often happens when bilinguals or multilinguals believe words in another language can better represent their thoughts (Proverbio et al., 2004) or would like to exhibit cultural closure to their interlocuters (Gafaranga, 2007). Although bilingual speakers often produce code-switches intentionally to achieve communicative or social goals, bilingual readers and listeners must instead comprehend language switches that are externally imposed by the linguistic input. Such switches present a unique challenge for the comprehension system because readers must integrate incoming information while adapting to a change in language. Consequently, an important question in bilingual psycholinguistics is whether the cognitive cost associated with language switching reflects the operation of language-control mechanisms or more general processes involved in generating and updating contextual expectations during language comprehension. From a language processing perspective, code-switching introduces a salient form of linguistic conflict, as readers must rapidly adjust to a change in language and reconfigure lexical access. However, language switching is only one type of mismatch that readers encounter. Even within a single language, inconsistencies at the semantic or discourse level require readers to detect conflicts between new information and their evolving mental representation of the text.

### Monolingual Text Processing and the Cognitive Cost of Inconsistency

Reading is an incremental and predictive process in which readers continuously generate expectations about upcoming linguistic input based on lexical, syntactic, and discourse-level information (Rayner, 2009). According to the predictive coding account, language comprehension is fundamentally anticipatory (Rao & Ballard, 1999). Rather than passively integrating incoming words, readers continuously generate probabilistic predictions about upcoming linguistic input based on lexical, syntactic, and discourse-level context. Under typical monolingual conditions, processing is facilitated when incoming input matches these expectations. However, violations of semantic or syntactic predictions reliably induce measurable cognitive costs, reflected in both behavioral and neural indices.

At the neural level, semantic incongruity robustly elicits the N400 component, a centro-parietal negativity peaking around 400ms after stimulus onset (Kutas & Hillyard, 1984). The amplitude of the N400 is modulated by contextual constraint: highly predictable words generate smaller N400 amplitudes, whereas semantically anomalous or low-probability words elicit larger responses. This pattern has been interpreted as reflecting the ease or difficulty of lexical-semantic integration within a developing sentence representation. Importantly, the N400 is graded by expectancy rather than categorical anomaly, suggesting that semantic processing is probabilistic and prediction-driven (Federmeier & Kutas, 1999; Lindborg et al., 2023).

Syntactic violations, in contrast, are often associated with the P600 component, a late posterior positivity linked to structural reanalysis and repair (Osterhout & Holcomb, 1992). While early research treated the N400 and P600 as markers of distinct semantic versus syntactic processes, more recent perspectives emphasize interactive and cascading processing mechanisms in which semantic and structural information jointly constrain comprehension (Schacht et al., 2014; Delogu et al., 2025).

Eye-tracking research complements ERP findings by revealing how these violations influence moment-to-moment reading behavior. For instance, Ahn et al. (2020) showed that bilingual readers exhibited processing costs during language switching in comprehension, while also demonstrating that language control during comprehension differs from that involved in language production. More recently, Schwartz et al. (2025) found that language switches produced longer reading times and altered eye-movement patterns, suggesting that both language-specific inhibitory control and domain-general cognitive control contribute to bilingual language comprehension. Disruptions to semantic or syntactic consistency increase first-pass fixation durations, regressions, and total reading times (Rayner, 2009). Such measures provide evidence that inconsistency incurs immediate processing costs and may trigger reanalysis. Crucially, these effects scale with contextual constraint: violations in strongly predictive contexts produce greater disruption than violations in weak contexts (Rayner & Well, 1996; White et al., 2005), consistent with predictive coding accounts of language comprehension.

Recent work incorporating computational measures such as word surprisal further supports this expectation-based framework. It has been shown that surprisal, the negative log probability of a word given its prior context, can modulate both ERP amplitudes and reading times (Chen et al., 2023). Words that are less predictable within a discourse context generate larger N400 effects and longer reading times. These findings reinforce the view that comprehension involves continuous probabilistic updating of expectations, and that cognitive cost may arise when incoming input deviates from these expectations.

Importantly, context accumulation plays a central role in shaping expectations (Nieuwland & Van Berkum, 2006). As discourse unfolds across multiple sentences, readers build increasingly constrained situation models. Violations occurring after extended contextual build-up should therefore generate larger conflict-driven effects (e.g., longer fixations and inflated N400s) than violations following minimal context (Frank et al., 2015; Luke & Christianson, 2016; Payne et al., 2015). Thus, in monolingual processing, cognitive effort increases not solely a function of local anomaly, but also a result of prior contextual investment.

Taken together, monolingual research establishes two critical principles: (1) semantic inconsistency generates robust cognitive cost, and (2) this cost is modulated by the strength of prior contextual expectations. Together, these findings suggest that processing difficulty is strongly influenced by readers’ evolving contextual expectations. However, bilingual language comprehension introduces an additional source of potential processing cost: changes in language itself. An important question, therefore, is whether the cognitive cost associated with code-switching reflects the same expectation-based mechanisms observed in monolingual comprehension, or whether switching between languages imposes an additional processing burden beyond contextual surprisal.

### Bilingual Language Processing and the Cognitive Cost of Inconsistency

Building on findings from monolingual reading, researchers have asked whether the processing costs observed during bilingual language comprehension arise from similar expectation-based mechanisms or from the need to manage two simultaneously active language systems. Different from monolingual readers, bilinguals possess two linguistic systems that can be co-activated during comprehension. A central question in bilingual psycholinguistics concerns whether these languages compete during processing and whether such competition produces measurable cognitive cost. Evidence from both behavioral and neural research suggests that bilingual lexical access is largely non-selective (Spivey & Marian, 1991; Shook & Marian, 2012). Even when reading in a single language, representations from the other language can become activated, particularly when orthographic or phonological overlap exists (Jouravlev et al., 2014; Zhang et al., 2022). Research has also revealed that such co-activation can create interference during bilingual language comprehension (Macnamara & Kushnir, 1971). Indeed, Altarriba et al. (1996) discovered that Spanish-English bilingual participants’ first fixation durations were longer on code-switched words than on non-switched words during reading, suggesting that there is a cost when processing code-switched linguistic information.

To explain how bilinguals successfully comprehend and produce language despite this parallel activation, researchers have proposed different mechanisms of bilingual language control. Activation-based accounts (e.g., BIA+) emphasize the role of relative activation and competition among lexical representations (Dijkstra & Van Heuven, 2002), whereas inhibition-based accounts propose that bilinguals actively suppress the non-target language to facilitate processing in the target language (Green, 1998).

In particular, comprehension of language switches may not require the same degree of active suppression because bilingual readers can use preceding linguistic context to anticipate the upcoming language. Thus, processing difficulty following a language switch may reflect violations of contextual expectations rather than solely the need to inhibit the previously active language.

The reason why such cost exists during the comprehension of code-switching remains relatively unclear, however. Evidence from monolingual reading research demonstrates that readers experience additional processing costs when incoming information violates stronger contextual expectations. In bilingual reading, this dynamic is further complicated by the presence of language switching. Here, mismatches may stem not only from semantic or discourse-level inconsistencies, but also from shifts in linguistic form, introducing an additional layer of unpredictability. The cost of switching languages may reflect the need to adjust the relative activation of the two languages after a switch is detected. Under inhibition-based accounts, the previously active language must be suppressed, whereas activation-based accounts suggest that both languages remain active and that processing difficulty arises from managing competing representations. This process might be the source of the increase in cognitive load. Another possibility is that the prediction or expectation of a specific set of semantic features, or even of a specific word (including its graphemic realization) is formed during language comprehension, and the cost of code-switching stems from encountering a word that is realized in an unexpected form/language. Consequently, an important theoretical debate concerns the origin of code-switching costs. Specifically, researchers have questioned whether processing difficulty arises because readers must shift between languages, or because language switches violate lexical and semantic expectations generated by the preceding context.

Moreno et al. (2002) used ERPs to investigate the origin of intra-sentential code-switching cost. The researchers proposed that if code-switching cost is incurred due to the realization of a switch in language, Late Positive Complex (LPC) effects should be observed in ERP data, as they are associated with sentence-level integration (Van Hell et al., 2015). On the other hand, if the cost is due to encountering an unexpected word, an inflated N400 response should be observed during code-switching comprehension, as the N400 is associated with semantic processing. In the experiment, English-Spanish bilinguals read English sentences containing both regular code-switching of words and code-switching of words in idioms. Results showed an LPC effect and a relative absence of typical N400 effect in regular code-switching sentences compared to idioms, suggesting that the cost of intra-sentential code-switching may originate more from the realization of an unexpected language switch. Similarly, a study by Proverbio et al. (2004) done with interpreters has found an additive N400 effect for semantically unexpected words with their language switched, compared with unexpected words alone. This finding supports the hypothesis that the change in language itself is the primary source of cognitive burden in code-switching.

More recently, however, Yacovone et al. (2021) conducted an ERP study with Spanish-English bilinguals, in which participants listened to Spanish and English sentences that contained either strong-fitting or weak-fitting code-switched words, similar to the design of Proverbio et al. (2004). Results showed a non-additive N400 effect for the two experimental manipulations, an LPC effect for code-switched words, and persistent negativity in code-switched words, which is associated with integration difficulty. The researchers argued that the cost for code-switching “can be understood within more general frameworks for understanding language comprehension” (p. 1). Therefore, the processing cost brought by code-switching could largely be due to the surprisal brought by an unexpected word or its meaning instead of the processing of the switch in language on the global level; the latter could arise from participants’ realization of the change in orthography, morphology, or other visual properties. Results from FitzPatrick and Indefrey (2014) and Liao and Chan (2016) also support the word-level hypothesis.

Despite considerable progress, studies of intra-sentential code-switching face an important methodological limitation. Because the language switch and the critical word occur simultaneously, readers’ expectations about language and lexical meaning are inherently confounded. Consider a sentence such as “Yesterday I bought a new…” In a monolingual context, readers may simultaneously predict both the upcoming language (e.g., English) and a semantically appropriate continuation (e.g., car). If the sentence instead continues with the Chinese word 车, the violation of language expectation and lexical expectation occurs simultaneously, making it difficult to determine whether processing costs arise because the language changed or because the predicted lexical form was not encountered. Consequently, it remains difficult to determine whether observed processing costs reflect the realization of a language switch itself or the violation of contextual expectations. Examining language switches between sentences provides a promising approach for disentangling these possibilities.

### Cost of Between-Sentential Code-Switching

A few studies have investigated code-switching between sentences. For instance, in Ibáñez et al. (2010), professional translators read sentences in their L1 (Spanish) and L2 (English) presented in a random order in a self-paced reading task, and the reading times of the first word of the sentences were recorded. These time measures indicated that there was indeed a cost when participants encountered a different language. Similarly, in Gullifer et al. (2013), Spanish-English bilingual participants read sentences alternating in the two languages and were asked to name a target word in the sentence that was either a cognate or a non-cognate in Spanish and English. Different from Ibáñez et al. (2010), results showed that cognates were named faster than non-cognates, and there were no effects of such inter-sentential code-switching.

More recently, Pérez and Duñabeitia (2019) examined inter-sentential code-switching cost using both behavioral and EEG measures. In their experiment, Spanish-Basque bilinguals listened to Spanish-only, Basque-only, and mixed-language paragraphs of speech, and returned two days after for a memory recall test. Although behavioral data showed no memory penalty for mixed-language speech, EEG results did find reduced spectral power in the beta band range for mixed-language paragraphs, indicating some level of neural processing cost for code-switching happening between sentences. The locus of the cost, however, was not discernable from the experimental design.

Because these previous studies have shown relatively mixed results, their potential limitations should be addressed. Although previous studies, such as Ibáñez et al. (2010), have advanced our understanding of bilingual reading processes using self-paced reading paradigms, such methods do not fully reflect natural reading, during which readers can extract information from upcoming words through parafoveal vision before directly fixating on them. Models of eye-movement control, such as the E-Z Reader model (Reichle et al., 1998), propose that lexical processing of the fixated word guides decisions about when to move the eyes while allowing limited preprocessing of upcoming words. Thus, eye-tracking provides a sensitive measure of incremental processing and contextual expectation during reading. Moreover, word naming and memory tests are also not accurate behavioral measures of cognitive load during inter-sentential code-switching, as the former fails to capture the cost incurred at the start of the sentences (as the named words were located in the middle), and the latter cannot reflect the online process of bilingual language comprehension. In addition, although none of the studies investigated inter-sentence switch costs using ERP, it is still important to note that ERP results, especially the N400, may not be a reliable indication of increased cognitive burden in between-sentence switches, as previous work has shown that N400 effects are larger earlier in sentences in semantically congruent sentences (Payne et al., 2015) and decrease as the sentence continues. Similarly, cloze norms become more consistent and reading times decrease as sentences progress (Luke & Christianson, 2016). As such, words at the beginnings of sentences tend to be maximally unpredictable/unexpected, and therefore generate large N400s naturally, irrespective of language change.

Collectively, these studies demonstrate that inter-sentential code-switching remains poorly understood. Existing paradigms either lack naturalistic measures of online reading or cannot determine whether observed costs reflect language switching itself or violations of discourse-level expectations. Consequently, an eye-tracking investigation that independently manipulates language switching and contextual build-up can provide important new evidence regarding the mechanisms underlying bilingual language comprehension.

## The Current Study

The primary objective of the current study was to distinguish processing costs attributable to language switching from those arising from contextual expectations built over preceding discourse. Making this distinction has important theoretical implications. If language-switching costs persist after contextual expectations are controlled, this would suggest that bilingual comprehension engages mechanisms associated with language control, such as reconfiguring the active language context or coordinating two simultaneously active language systems. In contrast, if processing costs are largely explained by violations of contextual expectations, then language switching may be understood within the same predictive framework that accounts for monolingual language comprehension, suggesting that bilingual readers rely primarily on general mechanisms of linguistic prediction and integration. By independently manipulating language switching and contextual build-up while recording readers’ eye movements, the present study aims to disentangle these competing accounts.

The present study aims to fill in some of the existing gaps in the current literature. First and most importantly, since most previous studies have examined intra-sentential code-switching cost, it is difficult to tease apart the two kinds of cognitive load imposed by code-switching: the cost of experiencing inconsistency in the global features of the language, and the cost of encountering inconsistency in word-level expectations. These expectations can be generated at varying grain sizes. Although exact lexical prediction is rare in normal reading (Luke & Christianson, 2016), it is possible, if the context is explicit. Indeed, classic ERP experiments create sentences that allow for high rates of predictability to compare to counterpart sentences with extremely unpredictable words swapped in for the predictable ones (e.g., *I take my coffee with cream and sugar/dog* [Kutas & Hillyard, 1980 ]). In fact, reader predictions about upcoming material can include orthographic or phonological features of upcoming words. Luke and Christianson (2012) reported that the transposed-letter confusability effect (e.g., Andrews, 1996; Perea & Lupker, 2003), whereby words with transposed letters are misread for correctly spelled words (e.g., *jugde* for *judge*), is significantly reduced when the word is read within connected text and is highly predictable. Similarly, Laszlo and Federmeier (2009) observed reduced N400 amplitudes in response to unexpected orthographic neighbors of highly predictable words compared to unexpected orthographically dissimilar words.

In the current study, we examined the cost of code-switching happening between sentences, and therefore separate the two kinds of potential cost. In this experiment, participants were primed with sentences in one language and code-switched to the other in a subsequent sentence. Some of the sentence strings had a consistent narrative, whereas other sentence strings consisted of sentences that were independent of each other. In the inconsistent condition, any word in the target sentence would be unexpected, regardless of the language that it is in. In the consistent narratives, although first words in upcoming sentences were not lexically predictable, they were consistent with the semantics of the previous sentence, and thus their semantic features were predictable in a graded fashion (cf. Luke & Christianson, 2016). The wordform, however, was always unpredictable in the code-switched condition, as the English and Chinese orthographies are visually dissimilar in virtually every way. Thus, we were able to separate the prediction of the upcoming language from the graded prediction of the upcoming word. If code-switching cost is due solely to the switch in language, there should be no difference in participants’ eye-movement patterns when they read sentence strings which have a consistent narrative and those when they read unrelated sentences. On the other hand, if code-switching cost consists of both the cognitive load of experiencing inconsistent language and an inconsistent wordform in the given context, participants should exhibit more difficulty reading sentence groups with a consistent narrative. Moreover, there should be an additive penalty for language switches in consistent narratives, and this should increase with more narrative priming.

## Experiment

### Methods

#### Participants

Sixty-two participants were recruited from the international student population of the University of Illinois, Urbana-Champaign (mean age = 22.98 years, females = 37). All participants were sequential L1 Mandarin Chinese-L2 English bilinguals, with an average age of acquisition of English = 7.23 years. Orthographically, among the participants, 49 used Simplified Chinese, the variety primarily used in mainland China, and 13 used traditional Chinese, the variety primarily used in other Mandarin-speaking regions of the world. The eye-tracking data of the two groups were analyzed separately and then combined. All participants completed a language background survey after the eye-tracking session.

#### Design

The experiment was a 2 × 2 × 2 repeated-measures design. The independent variables were: (1) whether the target sentence was preceded by one or three prime sentences; (2) whether the narrative of the sentence group was consistent or inconsistent; and (3) the direction of code-switching (from Chinese to English, from English to Chinese). Because Chinese and English target sentences were analyzed separately, this factorial design was applied independently within each target language. In addition to these factorial conditions, two supplementary no-switch baseline conditions (Chinese-only and English-only) were included. In these baseline conditions, participants read passages that remained in the same language throughout but contained an inconsistent narrative under the high-context (three-prime) condition. These baseline conditions were not part of the factorial design; rather, they served as controls to estimate the processing cost of narrative inconsistency in the absence of a language switch. They allowed us to isolate switch-specific effects from general discourse violation effects. The dependent variables were the first fixation duration (FFD), gaze duration (GD), and total reading time (TT) of each word in the code-switched final sentence.

#### Materials

Sixty groups of sentences were created as the stimuli, six groups for each of the 10 conditions (8 experimental, 2 control). Each sentence consisted of a subject, a predicate, an object, and a prepositional phrase. Each sentence group consisted of one or three prime sentences in one language, and the target sentence in the other language in conditions where the code-switching occurred. The narrative of the sentence group would either be consistent, where sentences followed a logical progression of events in a scene, or inconsistent, where each sentence in the sentence group was independent and unrelated to any of the other sentences. In consistent conditions, the first word of the next sentence is always the pronoun of the subject in the first sentence. In inconsistent conditions, we always alternate the gender of the subject of the next sentence, which is the first pronoun. Therefore, readers could detect a clear distinction between the two conditions at very early reading. Example materials can be found in Table 1. We used ChatGPT to norm the sentence groups. A few studies have used ChatGPT to generate stimuli for psycholinguistics experiments (Alzahrani, 2025) or engage in linguistics tasks (Vitevitch, 2025) and have found great resemblance between ChatGPT and human performance, suggesting AI as a valid and promising tool in experimental design and psycholinguistic research development. Specifically, we provided ChatGPT with the prime sentences in each sentence group, in both languages and both consistency manipulations, and asked ChatGPT to predict the first word of the upcoming sentence based on the given sentences. Results showed ChatGPT was able to correctly predict the upcoming word in the consistent condition, with an accuracy rate of 98% in English sentences and 97% in Chinese sentences. For the inconsistent narrative condition, ChatGPT incorrectly predicted almost every first word of the upcoming sentence (higher than 99% in both English and Chinese sentences), demonstrating that the sentences in the inconsistent narrative were indeed not correlated with each other in terms of meaning. Each sentence group also contained a yes/no comprehension question which asked about information in the sentence prior to the target sentence to keep participants engaged and paying attention to the materials. An additional 4 groups of sentences were also created as practice trials prior to the formal experiment trials, with structures identical to the experiment trials. The language background survey asked participants to report their age, gender, language use, and self-rated English proficiency on a scale of 1–10, along with their highest TOEFL score.

**Table 1:** Example Experiment Stimuli

		1 Prime Sentence	3 Prime Sentences
Consistent Narrative	Prime 1		Daniel joined the chess club this semester.
	Prime 2		He takes part in club activities every week.
	Prime 3	Ming visited the doctor at the school clinic.	He takes part in club activities every week.
	Target	他明天会吃药。(He will take some medicine tomorrow.)	他总是赢下棋局。(He often wins the game.)
Inconsistent Narrative	Prime 1		汤姆在手机上设置了闹钟。(Tom set the alarm on his phone.)
	Prime 2		她这周二去了电影院。(She went to the movie theater this Tuesday.)
	Prime 3	安娜不久前喂了猫。(Anna fed the cat a while ago.)	他每天都听这支乐队的歌。(He listens to this band every day.)
	Target	She ordered iced latte at the café.	She turned in the essay just now.

#### Apparatus

Eye movement data were collected using an SR Research EyeLink 1000 remote desktop eye tracker running SR Research Experiment Builder software. A chin rest and forehead rest were used to stabilize participants’ heads. All sentences were presented in black SimSun font (14 pt) on the computer monitor, which was approximately 56 cm away from the participants. All viewing was binocular, but data were recorded from only the right eye.

#### Procedure

Written informed consent was obtained from all participants prior to participation. Participants were informed about the nature of eye-tracking procedures and their right to withdraw at any time without penalty. The study was approved by the institutional review board. Each of them received $7 in cash in compensation for a 45-minute session. They were then calibrated on the eye tracker using a nine-point calibration procedure. Participants were asked to read silently for comprehension, and after a practice session consisting of four sentence groups, 10 blocks were presented in a randomized order. Each sentence would appear on the screen interpedently, and participants were asked to press a button on a game controller when they finish reading a sentence to proceed to the next sentence. Participants answered the comprehension question by pressing one of two buttons on the controller. Recalibration was performed between every block. After the eye-tracking session, participants answered the language background survey.

#### Data Analysis

Data analysis was conducted in R (R Core Team, 2026) using linear mixed effects models and multiple linear regression with the *lme4* (Bates et al., 2015) and *lmerTest* (Kuznetsova et al., 2017) packages. For linear mixed effects models, participant and item were entered as random effects, while the other experiment manipulations were entered as fixed effects. The R code for running the models is included in the Appendix as well as the OSF repository (link below). Due to differences in orthographies, word length, and word boundaries, target sentences in Chinese and English were also analyzed separately. Consequently, language switching directions were not directly compared across languages. Instead, within each language, we compared switch and no-switch conditions. The fixed effects included the number of preceding priming sentences (one vs. three), narrative consistency (consistent vs. inconsistent), and switch condition (switch vs. no-switch). The dependent variables were first fixation duration, gaze duration, and total reading time measured on the first three words of English target sentences or the first three characters of Chinese target sentences. These regions were selected as the areas of interest because early processing following a language switch was expected to be reflected in the initial portion of the target sentence.

#### Data Availability

De-identified eye-tracking datasets, along with analysis scripts, are available at https://osf.io/3pm8n/overview?view_only=61cf510684a942daa29966fe669617a6. Due to ethical and privacy considerations, raw data containing potentially identifiable information are not publicly shared but may be made available upon reasonable request and approval by the institutional review board.

## Results

### English Proficiency

Participants self-rated their L2 proficiency as an average score of 6.6 on a scale of 1–10 (with 10 as the highest). Of those who reported their highest TOEFL score (36 out of 62), the average score was 104.24 out of 120. Previous studies that correlated self-rated language proficiency with standardized language proficiency testing have shown that bilinguals were able to rate their own language proficiency accurately (Scholl et al., 2021). As suggested by the average TOEFL score, participants overall had a fairly advanced proficiency level of English.

### Descriptive Statistics of Eye Movements

Table 2 summarizes the means of the combined raw eye-movement data of the both the simplified and traditional Chinese readers. All numbers are in milliseconds. For the eye-movement measures, FFD is first fixation durations, GD is gaze durations, and TRT is total reading times. Descriptive data from the simplified and traditional Chinese reader groups individually are presented in the Appendix.

**Table 2:** Summary of Descriptive Statistics of Eye-Movement Data of Two Reader Groups Combined

Target	English					Chinese				
Switch	Yes				No	Yes				No
Consistency	Consistent		Inconsistent		Inconsistent	Consistent		Inconsistent		Inconsistent
Prime	1	3	1	3	3	1	3	1	3	3
*First IA*										
FFD	259.35	237.04	252.41	260.65	244.74	237.82	246.24	256.51	255.66	275.48
GD	332.43	282.39	338.17	333.86	334.21	308.69	300.98	352.56	320.98	346.70
TRT	276.32	154.75	368.02	285.03	260.17	211.16	140.58	298.51	198.25	195.75
*Second IA*										
FFD	245.95	241.30	240.47	253.57	249.09	234.85	253.51	245.62	258.40	246.72
GD	342.22	339.92	328.33	338.57	337.88	275.43	294.66	271.47	288.97	282.06
TRT	505.77	439.35	616.62	516.46	472.26	278.19	236.97	291.02	239.96	253.95
*Third IA*										
FFD	220.23	224.37	226.46	220.07	229.13	221.30	243.98	236.08	234.66	250.86
GD	248.59	245.14	252.23	240.69	261.32	242.04	278.97	255.47	267.51	274.89
TRT	303.18	299.54	394.45	325.27	295.26	345.96	275.02	322.78	274.21	291.22

*IA* Interest Area, *Prime* Number of preceding sentence

#### English Target Sentences

Due to the small sample size of the traditional Chinese readers, we combined the two reader groups to obtain a comprehensive view of all participants’ reading behavior. Separate analyses for traditional and simplified character readers can be found in the OSF repository.

**Fig. 1:**
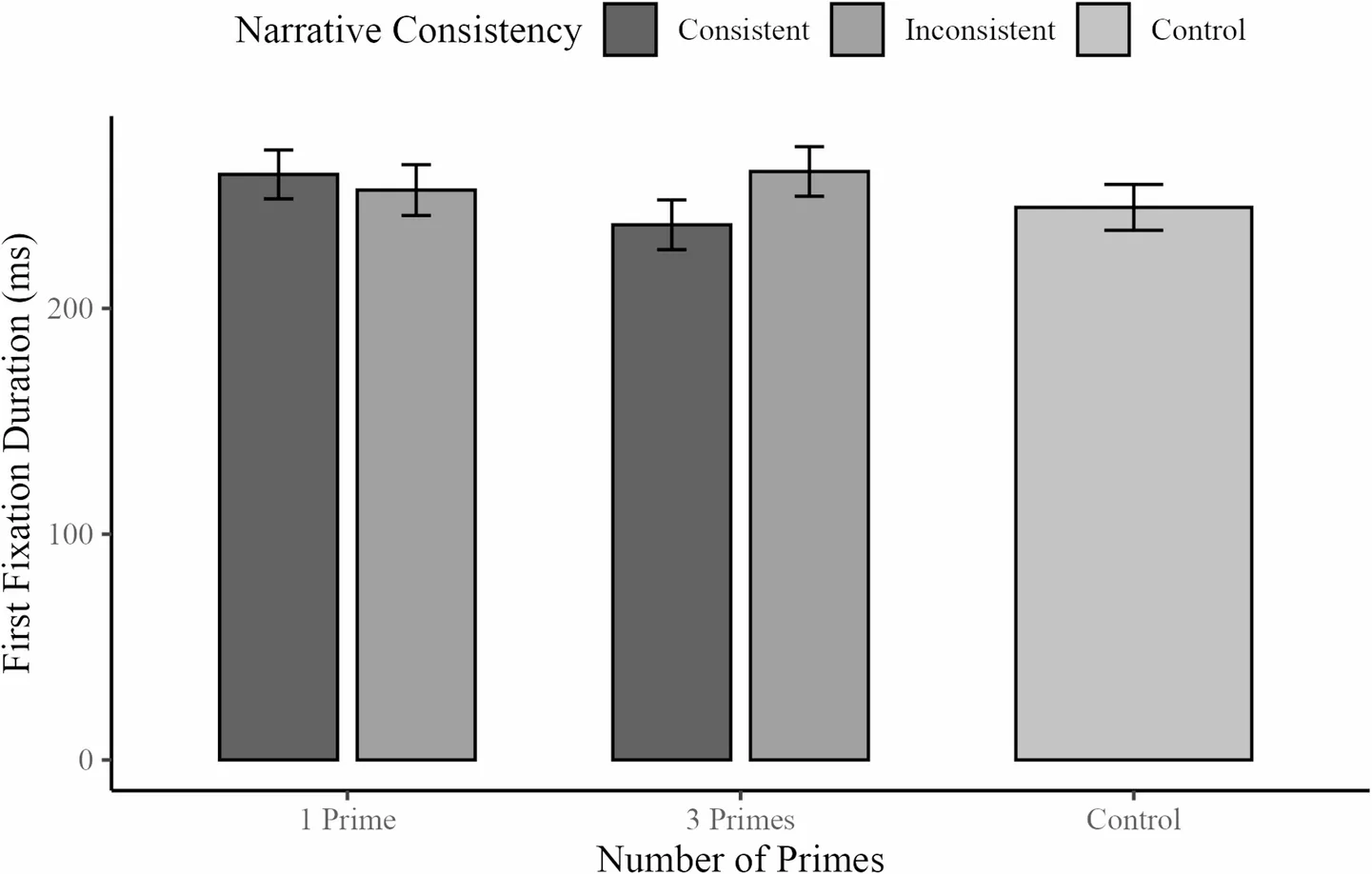
First Fixation Durations of the 1st word of English Target Sentences, Groups Combined

For English target sentences switched from Chinese, on the first word, we observed a significant main effect of prime sentence number (*b* = -23.02, SE = 10.29, *t* = -2.24, *p* = .026) and a significant prime × consistency interaction (*b* = 32.94, SE = 13.89, *t* = 2.37, *p* = .018) for FFD. The interaction is illustrated in Fig. 1. When the number of prime sentences increased, participants’ FFDs on the first word increased when there was a less consistent narrative preceding the switch. For GD, there was also a significant main effect of prime sentence number (*b* = -51.24, SE = 19.48, *t* = -2.63, *p* = .009). For TT, we observed significant main effects of prime sentence number (*b* = -121.43, SE = 24.45, *t* = -4.97, *p* < .001) and narrative consistency (*b* = 92.02, SE = 24.43, *t* = 3.77, *p* < .001).

On the second word, no effects of any kind were found for first fixation durations (FFD) or GD; for TT, we again found significant main effects of prime sentence number (*b* = -66.39, SE = 25.91, *t* = -2.56, *p* = .011) and narrative consistency (*b* = 110.93, SE = 25.89, *t* = 4.29, *p* < .001).

**Fig. 2:**
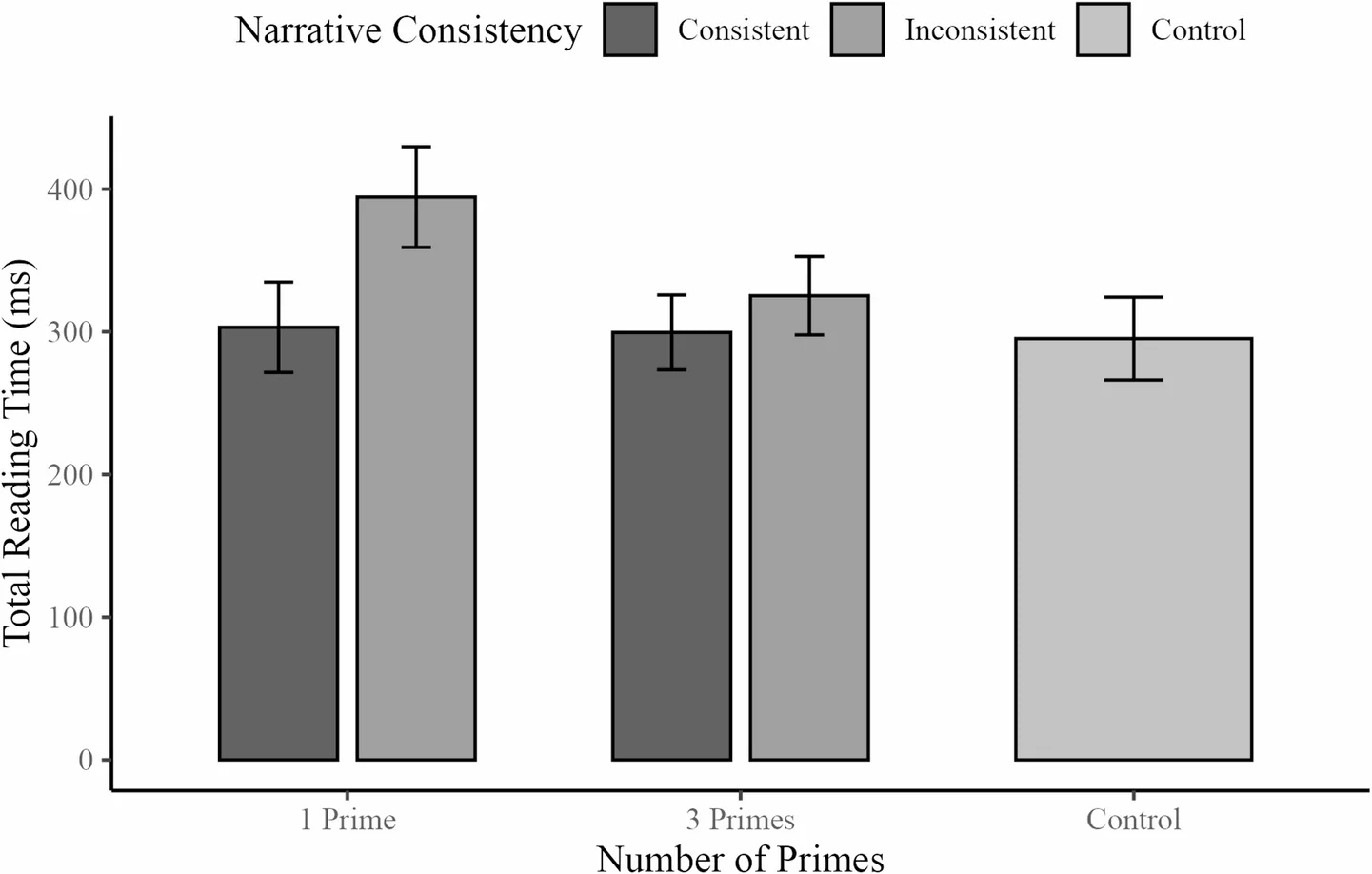
Total Reading Times of the 3rd word of English Target Sentences, Groups Combined

On the third word, there were no effects in FFD or GD. However, in TT, there was a significant main effect of consistency (*b* = 91.35, SE = 20.98, *t* = 4.35, *p* < .001), which was qualified by an interaction between prime sentence number and consistency (*b* = -65.71, SE = 29.66, *t* = -2.22, *p* = .027). The interaction is illustrated in Fig. 2. Similar to Fig. 1, TT on the third word decreased as the number of consistent sentences increased.

#### Chinese Target Sentences

Linear mixed effects models were also applied to the eye-movement data of the Chinese target sentences switched from English. For the first character, there was a main effect of language-switching on FFD (*b* = 29.71, SE = 12.14, *t* = 2.45, *p* = .015). Interestingly, when comparing the FFDs of sentences with and without a language switch, we found that participants spent more time in the baseline condition, where there were no language switches, than in the switch condition. Possible explanations will be discussed in the General Discussion. Although no effects were found for gaze durations (GD), there were significant main effects of prime sentence number (*b* = -69.71, SE = 22.18, *t* = -3.14, *p* = .002) and narrative consistency (*b* = 87.83, SE = 22.22, *t* = 3.95, *p* < .001) in TT.

On the second character, only a significant main effect was found for prime sentence number in TT (*b* = -41.12, SE = 20.38, *t* = -2.02, *p* = .044).

On the third character, in FFD, there were significant main effects of language-switching (*b* = 30.24, SE = 9.22, *t* = 3.28, *p* = .001) and prime sentence number (*b* = 21.80, SE = 9.34, *t* = 2.33, *p* = .020). Similarly, there were also main effects in GD of language-switching (*b* = 32.54, SE = 29.66, *t* = -2.22, *p* = .027) and prime sentence number (*b* = 36.10, SE = 12.50, *t* = 2.89, *p* = .004) and TT (language-switching, *b* = -54.75, SE = 19.58, *t* = -2.80, *p* = .005); prime sentence number, *b* = -70.75, SE = 19.57, *t* = -3.62, *p* < .001). There were no significant interactions.

Table 3 summarizes all the main effects and interactions found for each type of target sentences and both groups of readers. Although eye-movement measures starting from the fourth word have not been analyzed by linear mixed-effects models, linear regressions between eye-movement measures (FFD, GD, and TT) and word order have demonstrated that as word order increased, all eye-movement measures significantly decreased, suggesting that participants were able to recover from the processing cost as the reading proceeded. Code and results can be found in the OSF repository.

**Table 3:** Summary of Linear Mixed Effects Model Results

Target	English			Chinese		
*First IA*						
FFD	PS, PS×NC			LS		
GD	PS			N/A		
TT	PS, NC			PS, NC		
*Second IA*						
FFD	N/A			N/A		
GD	N/A			N/A		
TT	PS, NC			PS		
*Third IA*						
FFD	N/A			LS, PS		
GD	N/A			LS, PS		
TT	NC, PS×NC			LS, PS		

*FFD* First fixation durations, *GD* Gaze durations, *TRT* Total reading times, *LS* Language-switching, *PS* Prime sentence number, *NC* Narrative consistency

## Discussion

This study investigated the cause of processing cost during bilingual visual comprehension (reading). Previous research has suggested that the cognitive cost of language switching may arise from the surprisal of seeing another language in text (Van Hell et al., 2015), or from the unexpectedness of encountering a word that does not fit in terms of meaning or form with the reader’s expectations according to prior context (Yacovone et al., 2021). However, most previous studies focused on within-sentence language-switching, in which language and the specific word are switched at the same time. In the present study, we were able to tease apart these two factors by using between-sentence switching in the experimental materials. We treated language membership and word-level prediction as separable sources of potential cost by manipulating the presence of a language switch, the amount of prior semantic (discourse) information available before the target sentence, and the consistency of that discourse. In doing so, we embedded language switching within a broader framework of discourse-level predictive processing.

As shown in Table 3, the number of prime sentences and narrative consistency exerted greater effects on participants’ eye movements than language-switching itself. Aside from the third character in texts that switched to Chinese, language-switch costs were short-lived and primarily visible on the first word in early measures such as first fixation duration (FFD). Taken together, these results favor the word-level hypothesis: readers’ behavior was more negatively influenced by encountering an unexpected wordform that violated ongoing expectations than by the general surprisal of encountering an unexpected language. Importantly, this word-level surprisal was resolved quickly, whereas discourse-related manipulations produced effects in later measures. The pattern aligns with predictive accounts of language comprehension, in which readers continuously generate expectations at multiple representational levels—orthographic, lexical, and discourse—and incur processing cost when incoming input violates these probabilistic predictions.

A closer examination of Table 3 reveals systematic differences between early and later stages of processing. FFD is typically associated with early orthographic processing and lexical identification, whereas total reading time (TT) reflects later-stage semantic integration and discourse updating (Rayner et al., 2012). Language-switching effects were observed primarily in early measures (FFD of the first and third character) and were largely restricted to Chinese target sentences. In contrast, prime sentence number and narrative consistency predominantly affected TT, suggesting influence at the stage of discourse-level integration. This dissociation supports a layered account of processing: language-form violations trigger rapid, low-level adjustments, whereas discourse inconsistency affects later integration and global representation building.

The manipulation of prime sentence number provides insight into how contextual constraint modulates processing cost. Readers exposed to three prime sentences had more opportunity to accumulate semantic information and construct a coherent situation model compared to readers exposed to only one prime sentence. Within predictive frameworks, stronger contextual build-up should generate more constrained expectations about upcoming content. Consequently, violations occurring after extended context should produce larger prediction errors. The significant effects of prime sentence number are consistent with graded prediction accounts (Luke & Christianson, 2016), in which readers form increasingly strong expectations as discourse unfolds. When those expectations were violated, especially after multiple primes, integration difficulty increased, reflected in longer total reading times. The present findings are also consistent with recent evidence that code-switching costs are modulated by switch frequency. Salig et al. (2024) demonstrated that more frequently occurring code-switches in natural language elicited smaller comprehension costs than less frequent switch types, suggesting that bilingual readers learn statistical regularities governing code-switching behavior. Similarly, in the present study, participants in the one-prime condition experienced more frequent language switches throughout the experiment than those in the three-prime condition. Consequently, readers in the one-prime condition may have developed stronger expectations that a language switch could occur, reducing the processing cost associated with the switch.

Interestingly, readers appeared more affected by the number of prime sentences than by narrative consistency per se. Although the inconsistent narratives were deliberately constructed to create clear semantic discontinuities, participants had been informed about the two possible narrative patterns and thus may have adopted a strategy of actively monitoring discourse regularities. In longer (four-sentence) strings, readers had more time to detect and internalize patterns (whether consistent or inconsistent) thereby strengthening expectations about the upcoming target sentence. From a predictive processing perspective, this suggests that contextual constraint does not depend solely on semantic plausibility, but also on the stability of discourse patterns across sentences.

The interaction patterns further demonstrate how readers dynamically update expectations. For total reading times of the third word in English target sentences, there was an interaction between prime sentence number and consistency. As the number of prime sentences increased, participants spent less total reading time on inconsistent targets. We interpret this as a reduction in surprisal: after encountering consecutive inconsistent primes, readers adjusted their discourse-level expectations, thereby reducing the prediction error associated with another inconsistent continuation. In contrast, early measures (FFD of the first word) showed the opposite pattern: greater prime sentence number increased early disruption in inconsistent conditions. This suggests that initial contact with the target sentence activated strong top-down expectations derived from prior discourse, leading to transient cost when those expectations were challenged. However, during later integration stages, readers successfully incorporated the broader discourse pattern into their representation, reducing overall integration difficulty. Together, these results illustrate adaptive predictive updating: readers continuously recalibrate expectations based on accumulated context.

Comparing switches between languages, we found that participants were more disrupted when switching from English to Chinese than vice versa. Notably, in Chinese target sentences, participants sometimes spent more time in baseline (no-switch) conditions than in switch conditions. One interpretation, consistent with predictive accounts, is that language continuity itself becomes part of the discourse expectation. When no switch occurred, readers may have formed stronger expectations that the semantic content would also continue coherently in the same language. When that semantic expectation was violated, greater integration effort was required. In contrast, encountering a language switch may have signaled a contextual shift, prompting readers to adopt a more flexible predictive stance and thereby attenuating semantic surprisal. Another possible explanation involves global trial-level probabilities. Given that switch trials were more frequent than baseline trials, participants may have developed an expectation of switching at the experiment level. In this case, a baseline trial could itself constitute a probabilistic violation, producing short-lived increases in FFD. This interpretation further underscores the sensitivity of readers to distributional statistics and supports the view that prediction operates at multiple timescales—from local lexical probabilities to broader discourse and task-level expectations.

We did not observe robust language-switching effects in English target sentences when switching from L1 to L2. One possibility is that participants, as native Mandarin speakers, devoted greater cognitive resources to decoding English, their L2. If baseline processing load was already elevated, additional switch-related cost may have been less detectable. Alternatively, switching into L1 (Chinese) may produce stronger contrast effects because orthographic and structural properties differ markedly between English and Chinese. When the target language was native and visually distinct, early orthographic processing may have been especially sensitive to language-form change, producing observable FFD effects. These findings suggest that the orthographic and proficiency status of the target language modulates sensitivity to language switching, potentially explaining asymmetries reported in prior work (e.g., Costa & Santesteban, 2004). More broadly, they reinforce the idea that language-switch cost interacts with baseline processing demands and reader proficiency.

The present findings also revealed an asymmetry in language-switching costs, with greater processing difficulty observed when participants switched from English back to their dominant language, Mandarin. This pattern is consistent with the asymmetric switch costs commonly reported in bilingual language production, where switching into the dominant language often incurs greater costs than switching into the less dominant language (e.g., Meuter & Allport, 1999). Within inhibitory control accounts, this asymmetry is attributed to the stronger inhibition applied to the dominant language during use of the less dominant language, requiring greater effort to reactivate the dominant language when switching back. Although evidence for asymmetric switch costs has been considerably more limited in language comprehension than in production, the present findings suggest that similar mechanisms may also influence online bilingual reading. At the same time, because the current study did not directly manipulate inhibitory control, this interpretation should be considered tentative. Future work combining eye-tracking with neurophysiological measures or experimental manipulations of language dominance may help clarify the mechanisms underlying this asymmetry.

This study has several limitations. Word length and frequency were not tightly controlled across languages due to orthographic differences between English and Chinese. Future research could examine language pairs with more similar orthographic systems or incorporate more fine-grained lexical controls. Additionally, future studies could further disentangle language-level and discourse-level prediction by manipulating switch probability independently of semantic consistency. Another potential source of individual variability is participants’ habitual code-switching experience. Previous research has shown that bilinguals differ substantially in how frequently they switch languages and in the contexts in which switching occurs (Beatty-Martínez et al., 2020). Such experience may influence readers’ expectations regarding language changes and consequently modulate language-switching costs. Because the present study did not assess participants’ everyday code-switching habits or the typical direction of their language switches, we cannot determine whether these factors contributed to the observed effects. Future studies should incorporate measures of naturalistic code-switching experience to examine how interactional language use shapes online comprehension. Moreover, we calculated the skipping rates for each interest area in both the English and Chinese target sentences. For the English target sentences, the skipping rates were 41.32%, 8.00%, and 19.67% for the first, second, and third interest areas, respectively. For the Chinese target sentences, the corresponding skipping rates were 53.67%, 33.61%, and 24.01%. The relatively high skipping rates, particularly for the initial interest areas, may have reduced the sensitivity of the eye-tracking measures to detect language-switching effects and therefore represent a limitation of the present study. Future research should examine skipping and regression measures alongside traditional fixation-time measures to better understand their potential contribution to processing during language switching.

In conclusion, the results suggest that the cost of language-switching in between-sentence reading arises largely from the surprisal of encountering an unexpected wordform or semantic continuation rather than from the language switch itself. Language-form violations produced brief early disruptions, whereas discourse-level inconsistency and contextual constraint exerted more sustained influence on integration processes. The interaction between prime sentence number and narrative consistency demonstrates that readers dynamically adjust expectations based on accumulated context, consistent with predictive models of language comprehension. Thus, inter-sentential code-switching effects appear to be embedded within general mechanisms of discourse prediction and expectation updating, rather than reflecting a qualitatively distinct bilingual processing cost.

## Supplementary Information

Supplementary Material 1.
