# Supplementary material for: Language-Switching Cost in Chinese-English Bilingual Reading Comprehension: Evidence from Eye-Tracking
Source: J Psycholinguist Res. 2026 Oct 5;55(6):132. doi: 10.1007/s10936-026-10315-z (PMC13638688; doi:10.1007/s10936-026-10315-z)
Supplement: Supplementary file 1 [file 10936_2026_10315_MOESM1_ESM.pdf]

Appendix A

Code for running the linear mixed effects models

```
library(lme4)
library(lmerTest)

# English
data_English = data_ordered %>%
  filter(cs_direction == "CE" | cs_direction == "NE")
data_English$cs_direction = as.factor(data_English$cs_direction)
## first word
data_English_1 = data_English %>%
  filter(IA_order == 1)
### first fixation duration
ffd1_E = lmer(FirstFixationDuration ~ cs_direction + primenumber + consistency +
cs_direction : primenumber + cs_direction : consistency + primenumber : consistency +
cs_direction : primenumber : consistency + (1|Subject) + (1|TrialID), data = data_English_1,
REML = F)
summary(ffd1_E)
### gaze duration
gd1_E = lmer(GazeDuration ~ cs_direction + primenumber + consistency + cs_direction :
primenumber + cs_direction : consistency + primenumber : consistency + cs_direction :
primenumber : consistency + (1|Subject) + (1|TrialID), data = data_English_1, REML = F)
summary(gd1_E)
### total reading time
trt1_E = lmer(TotalReadingTime ~ cs_direction + primenumber + consistency + cs_direction
: primenumber + cs_direction : consistency + primenumber : consistency + cs_direction :
primenumber : consistency + (1|Subject) + (1|TrialID), data = data_English_1, REML = F)
summary(trt1_E)
## second word
data_English_2 = data_English %>%
  filter(IA_order == 2)
### first fixation duration
ffd2_E = lmer(FirstFixationDuration ~ cs_direction + primenumber + consistency +
cs_direction : primenumber + cs_direction : consistency + primenumber : consistency +
cs_direction : primenumber : consistency + (1|Subject) + (1|TrialID), data = data_English_2,
REML = F)
summary(ffd2_E)
### gaze duration
gd2_E = lmer(GazeDuration ~ cs_direction + primenumber + consistency + cs_direction :
primenumber + cs_direction : consistency + primenumber : consistency + cs_direction :
primenumber : consistency + (1|Subject) + (1|TrialID), data = data_English_2, REML = F)
summary(gd2_E)
### total reading time
trt2_E = lmer(TotalReadingTime ~ cs_direction + primenumber + consistency + cs_direction
: primenumber + cs_direction : consistency + primenumber : consistency + cs_direction :
primenumber : consistency + (1|Subject) + (1|TrialID), data = data_English_2, REML = F)
summary(trt2_E)
## third word
```

```
data_English_3 = data_English %>%
  filter(IA_order == 3)
### first fixation duration
ffd3_E = lmer(FirstFixationDuration ~ cs_direction + primenumber + consistency +
cs_direction : primenumber + cs_direction : consistency + primenumber : consistency +
cs_direction : primenumber : consistency + (1|Subject) + (1|TrialID), data = data_English_3,
REML = F)
summary(ffd3_E)
### gaze duration
gd3_E = lmer(GazeDuration ~ cs_direction + primenumber + consistency + cs_direction :
primenumber + cs_direction : consistency + primenumber : consistency + cs_direction :
primenumber : consistency + (1|Subject) + (1|TrialID), data = data_English_3, REML = F)
summary(gd3_E)
### total reading time
trt3_E = lmer(TotalReadingTime ~ cs_direction + primenumber + consistency + cs_direction
: primenumber + cs_direction : consistency + primenumber : consistency + cs_direction :
primenumber : consistency + (1|Subject) + (1|TrialID), data = data_English_3, REML = F)
summary(trt3_E)

# Chinese
data_Chinese = data_ordered %>%
  filter(cs_direction == "EC" | cs_direction == "NC")
data_Chinese$cs_direction = as.factor(data_Chinese$cs_direction)
## first word
data_Chinese_1 = data_Chinese %>%
  filter(IA_order == 1)
### first fixation duration
ffd1_C = lmer(FirstFixationDuration ~ cs_direction + primenumber + consistency +
cs_direction : primenumber + cs_direction : consistency + primenumber : consistency +
cs_direction : primenumber : consistency + (1|Subject) + (1|TrialID), data = data_Chinese_1,
REML = F)
summary(ffd1_C)
### gaze duration
gd1_C = lmer(GazeDuration ~ cs_direction + primenumber + consistency + cs_direction :
primenumber + cs_direction : consistency + primenumber : consistency + cs_direction :
primenumber : consistency + (1|Subject) + (1|TrialID), data = data_Chinese_1, REML = F)
summary(gd1_C)
### total reading time
trt1_C = lmer(TotalReadingTime ~ cs_direction + primenumber + consistency + cs_direction
: primenumber + cs_direction : consistency + primenumber : consistency + cs_direction :
primenumber : consistency + (1|Subject) + (1|TrialID), data = data_Chinese_1, REML = F)
summary(trt1_C)
## second word
data_Chinese_2 = data_Chinese %>%
  filter(IA_order == 2)
### first fixation duration
ffd2_C = lmer(FirstFixationDuration ~ cs_direction + primenumber + consistency +
cs_direction : primenumber + cs_direction : consistency + primenumber : consistency +
cs_direction : primenumber : consistency + (1|Subject) + (1|TrialID), data = data_Chinese_2,
REML = F)
```

```
summary(ffd2_C)
### gaze duration
gd2_C = lmer(GazeDuration ~ cs_direction + primenumber + consistency + cs_direction :
primenumber + cs_direction : consistency + primenumber : consistency + cs_direction :
primenumber : consistency + (1|Subject) + (1|TrialID), data = data_Chinese_2, REML = F)
summary(gd2_C)
### total reading time
trt2_C = lmer(TotalReadingTime ~ cs_direction + primenumber + consistency + cs_direction
: primenumber + cs_direction : consistency + primenumber : consistency + cs_direction :
primenumber : consistency + (1|Subject) + (1|TrialID), data = data_Chinese_2, REML = F)
summary(trt2_C)
## third word
data_Chinese_3 = data_Chinese %>%
  filter(IA_order == 3)
### first fixation duration
ffd3_C = lmer(FirstFixationDuration ~ cs_direction + primenumber + consistency +
cs_direction : primenumber + cs_direction : consistency + primenumber : consistency +
cs_direction : primenumber : consistency + (1|Subject) + (1|TrialID), data = data_Chinese_3,
REML = F)
summary(ffd3_C)
### gaze duration
gd3_C = lmer(GazeDuration ~ cs_direction + primenumber + consistency + cs_direction :
primenumber + cs_direction : consistency + primenumber : consistency + cs_direction :
primenumber : consistency + (1|Subject) + (1|TrialID), data = data_Chinese_3, REML = F)
summary(gd3_C)
### total reading time
trt3_C = lmer(TotalReadingTime ~ cs_direction + primenumber + consistency + cs_direction
: primenumber + cs_direction : consistency + primenumber : consistency + cs_direction :
primenumber : consistency + (1|Subject) + (1|TrialID), data = data_Chinese_3, REML = F)
summary(trt3_C)
```

Appendix B

Tables 3 and 4 present descriptive statistics of the eye-movement measures of the simplified Chinese reader group and the traditional Chinese reader group.

Simplified Chinese Readers

English Target Sentences

We analyzed the first fixation durations, gaze durations, and total reading times of each of the first three words of the English target sentences using linear mixed effects models. Due to the large number of conditions and measures, only the models with significant main effects and interactions will be shown in tables and figures.

For the first word of each target sentence, no significant effects were found for any of the independent variables for first fixation durations and gaze durations. For total reading times, there were significant main effects for prime sentence number (b = -128.26, SE = 27.08, t = -4.74, p < .001) and narrative consistency (b = 69.57, SE = 27.08, t = 2.57, p = .01).

The same was found for the second word; only significant main effects for prime sentence number (b = -80.26, SE = 28.29, t = -2.84, p < .01) and consistency (b = 109.40, SE = 27.08, t = 3.87, p < .001) were found for total reading times.

Figure 4

Interaction Between Prime Number and Narrative Consistency, 3rd Word, Simplified Chinese

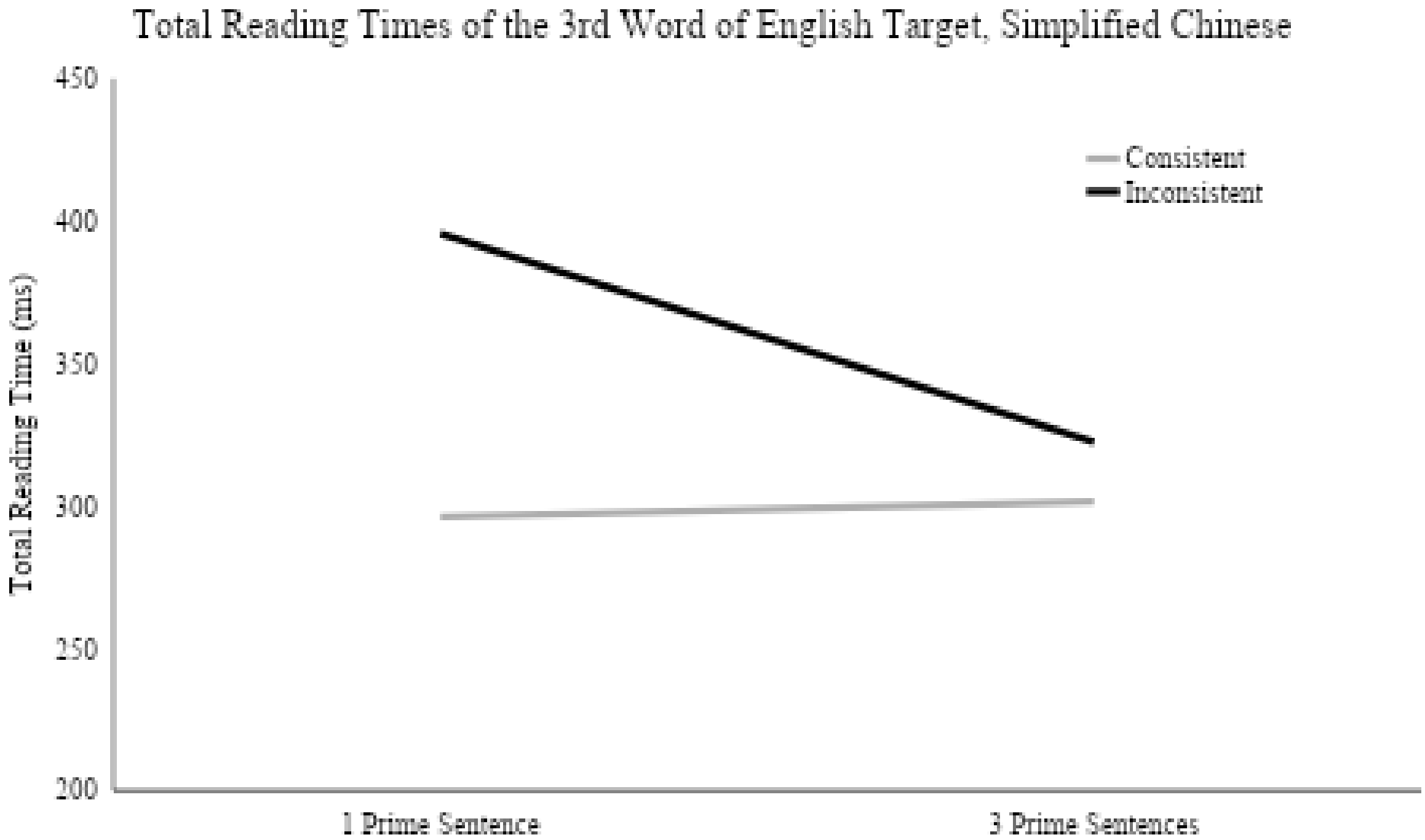

On the third word, similarly, no effects were found for first fixation durations and gaze durations; however, for total reading times, we observed a significant main effect for narrative consistency (b = 99.53, SE = 22.95, t = 4.34, p < .001) and an interaction between prime sentence number and consistency (b = -78.68, SE = 32.44, t = -2.34, p = .02). The interaction is illustrated in Figure 1. When the narrative context was inconsistent, participants spent less total reading time on third word in the 3-prime-sentence condition compared with that in the 1-prime condition. It is possible that participants were accustomed to the inconsistency in the input in the 3-prime condition and therefore experienced less surprisal when they encountered the target sentence, which was still inconsistent with the prior context and was also in another language.

Chinese Target Sentences

We analyzed the first fixation durations, gaze durations, and total reading times of each of the first three characters of the Chinese target sentences using linear mixed effects models. For the first character, we found a significant main effect of language-switching in first fixation durations (b = 29.32, SE = 12.28, t = 2.35, p = 0.02). No effects were found for gaze durations. Similar to English target sentences, for total reading times, we found main effects of prime sentence number (b = -79.93, SE = 25.66, t = -3.12, p = .002) and consistency (b = 94.95, SE = 25.68, t = 3.70, p < .001).

No significant effects were found for any of the eye-tracking measures on the second character.

On the third character, we also found a main effect of language-switching in first fixation durations (b = 34.01, SE = 9.93, t = 3.42, p < .001). For gaze durations, we found significant main effects of language-switching (b = 37.44, SE = 13.69, t = 2.74, p = .006) and prime sentence number (b = 32.42, SE = 13.81, t = 2.35, p = .019). Similarly, we found significant main effects of language-switching (b = -54.64, SE = 21.81, t = -2.51, p = .012) and prime sentence number (b = -69.50, SE = 21.80, t = -3.19, p = .001) for total reading times. No interactions of any kind were found for any of the eye-tracking measures.

Traditional Chinese Readers

English Target Sentences

Results of the traditional Chinese readers were similar to those of simplified Chinese readers. For English target sentences, no effects were found for the first word in first fixation durations. In gaze durations, we observed a significant main effect of prime sentence number (b = -76.51, SE = 35.91, t = -2.13, p = .034). For total reading times, we found a significant main effect of narrative consistency (b = 221.52, SE = 53.40, t = 4.15, p < .001).

No significant effects were found for any of the eye-tracking measures on the second word.

On the third word, we found a significant main effect of language-switching in first fixation durations (b = 40.43, SE = 17.33, t = 2.33, p = .020), and no effects of any kind for gaze durations and total reading times.

Chinese Target Sentences

Linear mixed effects models were also applied to the eye-tracking measures of the first three characters of the Chinese target sentences. For the first character, we observed a significant main effect of prime sentence number (b = 97.67, SE = 47.85, t = 2.04, p = .048) in first fixation durations. No effects of any of the conditions were found for gaze durations and total reading times.

No significant effects were found for any of the eye-tracking measures on the second character.

On the third character, we found a significant main effect of prime sentence number in first fixation durations (b = 63.18, SE = 25.15, t = 2.51, p = .013). Similarly, a significant main effect of prime sentence number was also found in gaze durations (b = 57.95 SE = 26.87, t = 2.16, p = .032). No significant effects were found for total reading times.

Table 4

Summary of Descriptive Statistics of Eye-Movement Data of Simplified Chinese Readers

Target	English					Chinese				
Switch	Yes				No	Yes				No
Consistency	Consistent		Inconsistent		Inconsistent	Consistent		Inconsistent		Inconsistent
Prime	1	3	1	3	3	1	3	1	3	3
First IA										
FFD	261.62	241.16	248.47	252.51	241.89	240.78	244.86	257.76	254.40	278.17
GD	336.74	292.67	338.42	331.05	336.57	315.38	301.99	358.92	322.53	352.27
TRT	287.82	159.20	357.02	281.89	254.24	340.68	159.57	334.73	224.36	225.72
Second IA										
FFD	244.15	238.85	239.31	254.74	254.79	232.96	255.79	247.36	257.05	248.66
GD	348.09	338.56	329.76	340.20	340.06	276.34	301.37	276.78	290.81	287.93
TRT	507.06	426.72	616.38	508.99	459.36	297.62	254.74	304.95	248.20	260.24
Third IA										
FFD	222.67	225.14	224.83	220.08	225.30	223.02	239.70	239.32	233.39	256.30
GD	249.64	244.58	252.01	241.46	257.00	246.36	279.73	258.61	270.62	284.29

TRT	296.13	301.51	395.57	322.37	284.19	357.62	287.85	334.54	279.63	302.79

NOTE: IA = Interest Area; Prime = number of preceding sentence

Table 5

Summary of Descriptive Statistics of Eye-Movement Data of Traditional Chinese Readers

Target	English					Chinese				
Switch	Yes				No	Yes				No
Consistency	Consistent		Inconsistent		Inconsistent	Consistent		Inconsistent		Inconsistent
Prime	1	3	1	3	3	1	3	1	3	3
First IA										
FFD	246.61	216.29	271.80	306.35	258.15	181.67	277.83	235.45	284.86	208.5
GD	308.33	230.61	336.98	349.68	323.13	181.67	277.83	244.91	284.86	208.5
TRT	210.07	128.60	431.59	303.19	294.55	41.69	30.87	85.94	47.91	23.17
Second IA										
FFD	256.00	254.80	246.91	246.98	218.96	248.73	236.67	232.97	266.72	234.86
GD	309.38	347.47	320.42	329.40	326.33	268.77	244.96	232.97	277.63	246.14
TRT	498.37	513.68	618.04	559.65	547.72	166.65	134.30	209.32	192.46	217.70
Third IA										

FFD	207.74	220.11	235.41	220.02	248.73	210.85	273.70	216.07	242.47	217.79
GD	243.22	248.27	253.41	236.69	283.41	215.93	273.70	236.05	248.32	217.79
TRT	343.93	287.92	388.02	342.07	359.96	279.00	200.91	253.77	242.96	223.56

NOTE: IA = Interest Area; Prime = number of preceding sentence
